# Expression of Endothelin-A-Receptor predicts unfavourable response to neoadjuvant chemotherapy in locally advanced breast cancer

**DOI:** 10.1038/sj.bjc.6601889

**Published:** 2004-06-29

**Authors:** P Wülfing, J Tio, C Kersting, B Sonntag, H Buerger, C Wülfing, U Euler, W Boecker, A H Tulusan, L Kiesel

**Affiliations:** 1 Department of Obstetrics and Gynecology, University of Münster, Münster, Germany; 2 Department of Pathology, University of Münster, Münster, Germany; 3 Department of Urology, University of Münster, Münster, Germany; 4 Department of Obstetrics and Gynecology, Klinikum Bayreuth, Bayreuth, Germany

**Keywords:** immunohistochemistry, preoperative, ET_A_R, ET_B_R, ET-1, chemoresistance

## Abstract

Endothelin-1 (ET-1) and its receptors (ET_A_R and ET_B_R), referred to as the endothelin (ET) axis, are overexpressed in breast carcinomas and appear to influence tumour growth and progression. The objective of this study was to determine the effect of expression of the ET axis in breast carcinomas on response to cytotoxic chemotherapy. The study included 44 patients with locally advanced breast cancer participating in a prospective phase III study evaluating high-dose neoadjuvant chemotherapy of epirubicin and cyclophosphamide. Expression of ET-1, ET_A_R and ET_B_R was determined by semiquantitative immunohistochemical analysis of breast cancer tissue from prechemotherapy tru-cut biopsies. Immunohistochemical staining was positive for ET-1 in 61.5%, for ET_A_R in 35% and for ET_B_R in 35.9% of breast carcinomas. Pathological response to chemotherapy was significantly decreased in ET_A_R-positive patients (*P*=0.002). In total, 50% of ET_A_R-positive patients as compared to 7.7% of ET_A_R-negative patients attained pathologically ‘no change’. Logistic regression confirmed ET_A_R as an independent predictive marker for pathological response (*P*=0.009). These data indicate that increased expression of ET_A_R in breast carcinomas is associated with resistance to chemotherapy. Determination of ET_A_R status may serve as a predictive marker for identifying patients less likely to be responsive to conventional chemotherapy.

Breast cancer is the most common cancer in women worldwide and distant metastases are the leading cause of breast cancer related death (Ferlay *et al*, 2000). Thus, adjuvant systemic therapy has become the standard therapy to destroy residual or disseminated tumour cells. Neoadjuvant chemotherapy has been developed to improve breast-conserving operability in locally advanced breast cancers. Results from the NSABP B-18 study confirmed that neoadjuvant and adjuvant chemotherapy are equally effective on locoregional disease in women with operable breast cancer. Moreover, this study demonstrated that clinical and pathological response to neoadjuvant chemotherapy is predictive of patient outcome (Fisher *et al*, 1997, 1998). However, the failure of a number of tumours to respond to treatment and the appearance of resistant tumour cell populations upon relapse of an originally responsive malignancy are still major impediments to successful chemotherapy. Currently, no tumour marker is available for clinical use in predicting chemotherapy response in breast cancer. It is thus an important goal in oncologic research to identify molecular markers to facilitate risk-adapted individual therapy concepts.

The endothelins (ETs) comprise three 21 amino-acid peptides, ET-1, ET-2, and ET-3, which are multifunctional peptides with diverse activity (Yanagisawa *et al*, 1988). ET-1 is expressed primarily by endothelial, vascular smooth muscle and epithelial cells, whereas ET-2 and ET-3 are expressed in kidney epithelial cells and in gastrointestinal stromal cells (Levin, 1995; Nelson *et al*, 2003). Of the three ET isoforms, ET-1 has been the most extensively studied, and appears to be the most important isoform in cancer pathophysiology (Grant *et al*, 2003; Nelson *et al*, 2003). ET-1 expression is increased in several human malignancies including ovarian, prostate and colorectal cancer (Nelson *et al*, 1996, 2003; Salani *et al*, 2000; Asham *et al* 2001). Upregulation of ET-1 occurs in response to cell activation and is induced by various stimuli such as hypoxia, growth factors, and cytokines (Kourembanas *et al*, 1991). ET-1 effects are mediated through two subtypes of G protein-coupled receptors, ET_A_R and ET_B_R. ET_A_R binds ET-1 and ET-2 with high affinity and ET-3 with low affinity, whereas ET_B_R is nonselective with equal affinity for the three subtypes (Rubanji and Polokoff, 1994). It has been shown that ET-1 competitively binds to the ET_A_- and ET_B_-receptor, although ET_A_R is the dominant receptor (Bagnato *et al*, 1995, 1999). The complex of ET-peptides and ET receptors is referred to as the ‘ET axis’ (Nelson and Carducci, 1999). With respect to ET receptors, predominantly ET_A_R mediates tumour-associated functions, whereas there is less evidence for ET_B_R-dependent tumour-related functions (Grant *et al*, 2003; Nelson *et al*, 2003). Engagement of ET_A_-receptor by ET-1 triggers tumorigenesis and tumour progression by activation of tumour proliferation, invasion, angiogenesis, and inhibition of apoptosis (Pedram *et al*, 1997; Bagnato and Catt, 1998; Bagnato *et al*, 1999; Eberl *et al*, 2000b; Salani *et al*, 2000; Rosano *et al*, 2001; Del Bufalo *et al*, 2002). There is also evidence for an autocrine and/or paracrine mechanism of action of ET-1 including angiogenesis-promoting effects in malignant tissues. It has been shown that ET-1 induces endothelial cell growth through ET_B_R and exerts mitogenic effects on vascular smooth muscle cells and pericytes through ET_A_R (Bek and McMillen, 2000; Salani *et al*, 2000). Moreover, activation of ET_A_R by ET-1 stimulates the production of VEGF, which in turn induces endothelial cell proliferation and vascular permeability by increasing the levels of HIF-1*α* (Spinella *et al*, 2002). In addition to its mitogenic effects, ET-1 has also been found to contribute to tumour growth by protecting tumour cells from apoptosis (Eberl *et al*, 2000b). Increased ET_A_R expression has been demonstrated in malignant tissue in several cancer types including ovarian, prostate and colorectal cancer, whereas in normal tissue from these sites ET_B_R predominates (Nelson *et al*, 1996; Bagnato *et al*, 1999; Ali *et al*, 2000).

Studies have investigated expression of ET-1 in human breast carcinoma by applying radioimmunoassay (Yamashita *et al*, 1991; Yamashita *et al*, 1995), immunohistochemistry (Kojima and Nihei, 1995; Alanen *et al*, 2000), and quantitative RT–PCR (Alanen *et al*, 2000). Consistent with other reports on altered ET axis in breast cancer (Yamashita *et al*, 1991; Alanen *et al*, 2000), we have previously demonstrated an increased ET-1, ET_A_R, and ET_B_R expression in human breast carcinomas (Wülfing *et al*, 2003). In our series, overexpression of ET-1, ET_B_R and especially of ET_A_R was associated with clinicopathological parameters that characterise aggressive types of breast cancer and indicated a poor outcome, whereas other studies failed to confirm such correlations. In view of the above findings, the ET axis and especially ET_A_R has been proposed as a potential target for anticancer therapy (Bagnato *et al*, 2002; Pirtskhalaishvili and Nelson, 2002; Rosano *et al*, 2003).

Various biological markers are currently being investigated as predictors of chemotherapy response in breast cancer. Such factors may be used in the selection of neoadjuvant or adjuvant chemotherapeutic treatment. Indeed, biological markers with confirmed prognostic or predictive impact could also serve as targets for therapeutic compounds to improve current breast cancer therapies. Thus, the objective of the present study was to investigate the predictive value of the ET axis for response to neoadjuvant chemotherapy in patients treated for locally advanced breast cancer. As the vast majority of studies in the literature dealing with ET expression in malignancies showed the predominant role of the ET-1 isoform and the ET_A_-receptor, this study focused on the expression of these markers.

## PATIENTS AND METHODS

### Patients

A total of 44 patients diagnosed with locally advanced breast cancer (T_2–4_N_0–2_M_0_) participating in a prospective randomised phase III study evaluating a high-dose neoadjuvant chemotherapy consisting of epirubicin (Pharmacia GmbH, Erlangen, Germany) and cyclophosphamide (Baxter, Frankfurt, Germany) were included in the study (Euler *et al*, 2002). Patients were diagnosed and treated at the Department of Obstetrics and Gynecology, Klinikum Bayreuth, Germany, between August 1997 and March 2002. Their median age was 51 years (range, 29–66 years). None of the patients had objective skin inflammation or oedema. On first presentation, a tru-cut biopsy was performed to confirm the diagnosis histologically. Initial staging was comprised of clinical examination, bilateral mammography, bilateral breast sonography, sonography of the axillary region, chest X-ray, liver sonography, and bone scintigraphy. Table 1

**Table 1:** Characteristics of patients and tumours at the time of diagnosis (prior to neoadjuvant chemotherapy)

No. of patients	44
*Age* (years)	
Median (range)	51 (29–66)
	
*Menopausal status*	
Premenopausal	15 (34.9%)
Postmenopausal	28 (65.1%)
Unknown	1
	
*Tumour size*	
T2	39 (90.7%)
T3	3 (7%)
T4	1 (2.3%)
Unknown	1
	
*Lymph node status*	
N0	24 (57.1%)
N1	18 (42.9%)
Unknown	2
	
*Grading*^a^	
I	1 (2.7%)
II	12 (32.4%)
III	24 (64.9%)
Unknown	7
	
*Histology*	
Ductal	29 (65.9%)
Lobular	11 (25%)
Other	4 (9.1%)
	
*Oestrogen receptor*	
Negative	9 (21.4%)
Positive	33 (78.6%)
Unknown	2
	
*Progesterone receptor*	
Negative	12 (28.6%)
Positive	30 (71.4%)
Unknown	2
	
*Her-2/neu*	
Negative	29 (70.7%)
Positive	12 (29.3%)
Unknown	3
	
*Ki67*	
<18%	17 (41.5%)
⩾18%	24 (58.5%)
Unknown	3
	
*p53*	
Normal	28 (68.3%)
Mutation	13 (31.7%)
Unknown	3

a Grading of surgical resection specimens postchemotherapy; not applicable in seven patients.

lists the results of the clinically assessed pretherapeutic tumour size and lymph node status.

### Tumour samples

Prechemotherapy tissue samples obtained by tru-cut biopsy were processed by formaldehyde fixation and paraffin embedding. Oestrogen receptor (ER) and progesterone receptor (PgR) status, p53, Her-2/neu and Ki67 were assessed immunohistochemically. Analyses were performed by Dianon Systems, Inc. (Stratford, CT, USA), and Molecular Oncology International P.S. (Seattle, WA, USA) using scoring criteria suggested by these laboratories. Oestrogen receptor and PgR status were considered positive if ⩾10% of tumour cells stained positive. High proliferative activity was defined as ⩾18% of tumour cell nuclei stained with monoclonal antibodies (AB) against the Ki-67 antigen. Her-2/neu expression and p53 mutation were considered positive if ⩾10% of infiltrating tumour cells stained positive with at least moderate intensity. Details are given in Table 1.

### Treatment protocol

The study protocol was approved by the institutional review board (ethical committee, University of Nürnberg-Erlangen, Germany), and their informed consent was obtained. Neoadjuvant chemotherapy consisted of epirubicin (120 mg m^−2^) and cyclophosphamide (600 mg m^−2^) per cycle. Cycles were repeated every 2 weeks (arm A) or every 3 weeks (arm B) (21 out of 44 patients were randomised for arm A, 23 out of 44 for arm B). For patients in arm A, chemotherapy was followed by 5 *μ*g kg^−1^ day^−1^ GM-CSF (AMGEN, München, Germany) s.c. or i.v. on days 2–12 after each cycle. For arm B, supportive GM-CSF was required if leucocytes were <2000 *μ*l^−1^. The sizes of primary tumours and of axillary lymph nodes, when applicable, were measured every cycle by palpation and by the same clinician using a calliper. To assess the clinical response, changes in the product of the two largest diameters recorded as baseline and at the end of chemotherapy prior to surgery were measured. Additionally, tumour size was evaluated using sonography and mammography. Clinical response to chemotherapy was classified according to the criteria of the International Union Against Cancer (UICC) as follows: (i) complete response (CR), disappearance of all clinical signs of disease; (ii) partial response (PR), ⩾50% decrease in tumour size; (iii) no change (NC), clinically <50% decrease or <25% increase in tumour size; (iv) progressive disease (PD), ⩾25% increase in tumour size or appearance of new lesions (Hayward *et al*, 1977).

At 2 or 3 weeks after the third cycle of chemotherapy surgery was planned. Patients who showed any clinical response to the treatment (*n*=40) underwent breast-conserving surgery and irradiation of the residual breast (60 Gy delivered in 6 weeks). In nine of these patients, reconstruction of the breast had to be performed concomitantly (latissimus dorsi flap). Four patients without any regression of the tumour had a modified radical mastectomy.

Pathological response to preoperative chemotherapy was categorised semiquantitatively into four groups, according to the literature (Sinn *et al*, 1994): no effect (=grade 0); resorption and tumour sclerosis (=grade 1); minimal residual invasive tumour <0.5 cm (=grade 2); residual noninvasive tumour only (=grade 3); no tumour detectable (=grade 4). The final pathological response was designated as follows: grade 0, NC; grade 1–3, PR; grade 4, CR. Patients with pathological response to neoadjuvant chemotherapy (CR or PR) postoperatively received a fourth cycle of high-dose epirubicin and cyclophosphamide (EC) and subsequently two cycles of a cyclophosphamide-, methotrexate-, 5-fluorouracil (CMF)-containing regimen (methotrexate: medac, Hamburg, Germany; 5-fluorouracil: GRY-Pharma, Kirchzarten, Germany). In total, 12 cycles of 5-FU (2 g m^−2^, weekly) and four cycles of Taxol (Bristol-Meyers-Squibb, München, Germany) (Taxol=175 mg m^−2^, every 3 weeks) were given to pathological nonresponders (Klaassen *et al*, 1998).

### Preparation of surgical resection specimens

The surgical resection specimens at breast-conserving therapy or mastectomy were examined and cut up fresh.

Appropriate tissue blocks were routinely processed, fixed in formalin, and embedded in paraffin. Serial 3-*μ*m sections were cut and stained with haematoxylin and eosin. Tumour size (pT classification) was measured macroscopically on stained serial sections containing the largest tumour specimen, or estimated on the basis of a sequential series of slides. The cases were classified according to the TNM classification (UICC, 5th edition). Tumour grade was also assigned based on the UICC criteria, dividing tumours into grade I (well differentiated), grade II (moderately differentiated), and grade III (poorly differentiated). Pathological response was determined as described above. All cases were reviewed by two experienced pathologists.

### Immunohistochemistry for ET-1, ET_A_R, and ET_B_R expression

Since we wanted to investigate whether the ET axis has a predictive value for chemotherapy response, immunohistochemical analysis was carried out on paraffin-embedded tru-cut prechemotherapy biopsies. In total, 3-*μ*m sections from 44 breast cancer samples were mounted on Polysine microslides. For antigen retrieval, dewaxed and rehydrated sections were immersed in Reveal buffer (BioCarta, Hamburg, Germany) and boiled in a pressure cooker (103 kPa/15 psi for 5 min). After blocking nonspecific binding sites, sections were immunoreacted with primary AB over night at 4°C. Primary AB were directed at Endothelin-1 (Affinity BioReagents, Golden, USA, diluted 1 : 500), Endothelin-A-Receptor, ET_A_R, and Endothelin-B-Receptor, ET_B_R (both from Alexis, Grünberg, Germany, diluted 1 : 400). Sections were then treated for 10 min with methanol containing 0.6% H_2_O_2_ to quench endogenous peroxidase. Bound primary mouse AB to Endothelin-1 was detected using DAKO Mouse-EnVision-HRP. Bound primary sheep AB to ET_A_R and ET_B_R were detected using DAKO Rabbit-EnVision-HRP, via bridge AB rabbit-anti-sheep (Dianova, Hamburg, Germany, 1 : 50). HRP label was visualised with NovaRed substrate kit (Vector Laboratories, Burlingame, CA, USA). Prostate cancer tissue known to express ET_A_R and smooth muscle tissue with ET_B_ receptor activity served as positive controls.

Negative controls with omission of primary AB were included. After nuclear counterstaining with haematoxylin, cytoplasmic immunostaining intensity was categorised semiquantitatively into four groups as described previously (Wülfing *et al*, 2003): negative (score 0): no staining at all; weakly positive (score 1+): faint/barely perceptible cytoplasmic staining in the majority of the tumour cells; moderately positive (score 2+): a moderate cytoplasmic staining in the majority of the tumour cells and strongly positive (score 3+): a strong cytoplasmic staining of the majority of the tumour cells. The final score was designated as negative or positive as follows: score of 0–1, negative; and score of 2–3, positive.

### Data analysis

Staining results were evaluated semiquantitatively in a blind manner. Statistical analysis was performed using SPSS 10.0 statistical software. Correlations between ET expression in breast carcinomas and clinical or pathological response to neoadjuvant chemotherapy were tested by cross-tables applying *χ*^2^ test. Also, expression of ET-1, ET_A_R and ET_B_R was correlated to each other and to classic prognostic factors with use of *χ*^2^ test. For the analysis of response prediction, clinical response and pathological response were divided into two categories: ‘response’ [CR+PR] and ‘nonresponse’ [NC]. Then associations between expression of the ET axis and response to chemotherapy were tested with logistic regression model with the tested factors regarded as continuous variables. The level of significance was *P*⩽0.05.

## RESULTS

### Immunohistochemical ET-1-, ET_A_R-, and ET_B_R-expression

**Figure 1:**
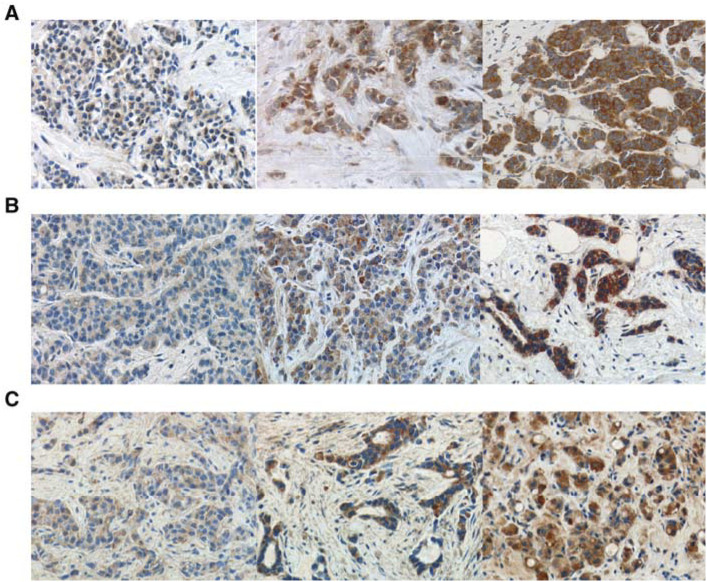
Representative immunohistochemical staining patterns for ET-1 (**A**), ET_A_R (**B**) and ET_B_R (**C**) in breast carcinomas. For each marker, a sample with weak (left), moderate (middle), and strong (right) cytoplasmic immunostaining is shown.

Immunolabelling for ET-1, ET_A_R, and ET_B_R presented as homogenous cytoplasmic staining. Intensity of ET-1, ET_A_R, and ET_B_R staining among different tumours varied from complete absence of staining to strong diffuse staining. Moderate or strong staining intensity defined as ‘positive’ immunoreaction was present for ET-1 in 24 out of 39 (61.5%), for ET_A_R in 14 out of 40 (35.0%), and for ET_B_R in 14 out of 39 (35.9%) evaluable breast carcinomas (Figure 1

). Strong stromal immunostaining was frequently detected in ETR-positive but not in ETR-negative cases. We observed a close and significant concordance between expression of ET_A_R and ET_B_R (*P*=0.013) and between ET_A_R and ET-1 (*P*=0.021) in breast cancers. No significant association between expression of ET-1 and ET_B_R (*P*=0.173) was found, although ET-1-positive tumours showed a trend towards ET_B_R positivity (Table 2

**Table 2:** Significance of the correlations between various factors given as *P*'s (*χ*^2^-test)

	**ET_A_R^a^**	**ET_B_R^a^**	**ER^b^,^d^**	**PgR^c^,^d^**	**Her-2/neu^e^**	**Ki67^d^**	**p53^e^**
ET-1^a^	0.021	0.173	0.031	0.006	0.630	0.258	0.630
ET_A_R^a^		0.013	0.145	0.184	0.416	0.045	0.416
ET_B_R^a^			0.219	0.270	0.459	0.415	0.351
ER^b^,^d^				<0.001	0.245	0.008	0.695
PgR^c^,^d^					0.865	0.001	0.698
Her-2/neu^e^						0.169	0.553
Ki67^d^							0.790

a Immunohistochemistry given as staining intensity.

b ER=oestrogen receptor status.

c PgR=progesterone receptor status.

d Immunohistochemistry given as percentage of positively stained cells (ER and PgR, ⩾10% of tumour cells stained positive; Ki67, ⩾18% of tumour cell nuclei stained).

e Immunohistochemistry given as staining index (⩾10% of tumour cells stained positive with at least moderate intensity).

).

#### Correlation between expression of ET axis and clinical and tumorbiological factors

The relationship among the expression of the ET axis and clinical characteristics of the breast carcinomas prior to chemotherapy is depicted in Table 3

**Table 3:** Relationship among ET-1, ET_A_R, and ET_B_R positive tumours and clinical/biological characteristics of breast carcinomas prior to neoadjuvant chemotherapy

	**ET-1 positive (*n*=39)**	***P***	**ET_A_R-positive (*n*=40)**	***P***	**ET_B_R-positive (*n*=39)**	***P***
*T*						
T2	19/34 (55.9%)	0.232	11/35 (31.4%)	0.190	12/34 (35.3%)	0.414
T3	3/3 (100%)		2/3 (66.7%)		1/3 (33.3%)	
T4	1/1 (100%)		1/1 (100%)		1/1 (100%)	
Missing	1		1		1	
						
*N*						
N0	12/24 (50%)	0.082	7/24 (29.2%)	0.391	7/22 (31.8%)	0.715
N+	11/14 (78.6%)		6/14 (42.9%)		6/16 (37.5%)	
Missing	1		2		1	
						
*G*^a^						
G1	—	0.281	—	0.436	1/1 (100%)	0.157
G2	5/10 (50%)		2/10 (20%)		2/11 (18.2%)	
G3	15/22 (68.2%)		9/23 (39.1%)		9/21 (42.9%)	
Missing	7		7		6	

a Grading of surgical resection specimens postchemotherapy.

. We observed no significant correlation between expression patterns of the ET axis with tumour size, lymph node involvement, or histological grading. Comparison between ET axis and tumorbiological factors revealed a strong inverse relationship between ET-1 expression and the steroid hormone receptor status (ER, *P*=0.031; PgR, *P*=0.006) (Table 2). Both steroid hormone receptors correlated positively with each other (*P*<0.001). ET_A_R expression, ER- and PgR-status were inversely correlated with proliferation rate as assessed by Ki67.

### Response to neoadjuvant chemotherapy

#### Treatment activity

All 44 investigated patients completed treatment according to the study protocol. The clinical response could not be determined in one patient (2.3%). At the end of the chemotherapy administration, eight patients (18.6%) attained a clinical CR (cCR) and 17 patients (39.5%) attained a clinical PR (cPR), for an overall clinical response rate of 58.1%. In total, 18 patients (41.9%) showed clinically NC (cNC), and no patient progressed. Information on pathological response was available for all patients. One patient (2.3%) was found to have a pathological CR (pCR) and 31 patients (70.5%) had a pathological PR (pPR). Thus, the overall pathological response rate was 72.7%. In all, 12 patients (27.3%) showed pathological NC (pNC).

#### Relationship between expression of ET axis and clinical response

Table 4

**Table 4:** Clinical response to neoadjuvant chemotherapy with respect to expression of ET-1, ET_A_R, and ET_B_R

	**Clinical response**					
	***n*^a^**	**CR^b^**	**PR**	**NC**	***P*^1^**	***P*^2^**
*ET-1*						
Negative	15	20% (3/15)	46.7% (7/15)	33.3% (5/15)		
Positive	23	21.7% (5/23)	39.1% (9/23)	39.1% (9/23)	0.897	0.717
						
*ET*_*A*_*R*						
Negative	26	23.1% (6/26)	50% (13/26)	26.9% (7/26)		
Positive	13	15.4% (2/13)	30.8% (4/13)	53.8% (7/13)	0.255	0.098
						
*ET*_*B*_*R*						
Negative	25	20% (5/25)	40% (10/25)	40% (10/25)		
Positive	13	23.1% (3/13)	38.5% (5/13)	38.5% (5/13)	0.976	0.604

a One patient missing in evaluation for clinical response.

b CR=complete response; PR=partial response; NC=no change. *P*^1^=CR *vs* PR *vs* NC (*χ*^2^ test). *P*^2^=response (CR+PR) *vs* nonresponse (NC) (*χ*^2^ test).

shows the clinical response to chemotherapy stratified with respect to the ET-1-, ET_A_R-, and ET_B_R-status. In total, 53.8% of ET_A_R-positive patients showed cNC as compared to 26.9% of ET_A_R-negative patients. Similarly, overall response to chemotherapy was more common in ET_A_R-negative than in ET_A_R-positive patients (73.1 *vs* 46.2%, respectively), although this difference did not reach statistical significance (*P*=0.098). No correlation was observed between ET-1 and ET_B_R-status and clinical response rate.

#### Relationship between expression of ET axis and pathological response

Differential expression of the ET axis with respect to pathological response is summarised in Table 5

**Table 5:** Pathological response to neoadjuvant chemotherapy stratified for ET-1, ET_A_R, and ET_B_R expression

	**Pathological response**					
	***n***	**CR^a^**	**PR**	**NC**	***P*^1^**	***P*^2^**
*ET-1*						
Negative	15	—	86.7% (13/15)	13.3% (2/15)		
Positive	24	4.2% (1/24)	62.5% (15/24)	33.3% (8/24)	0.245	0.164
						
*ET*_*A*_*R*						
Negative	26	3.8% (1/26)	88.5% (23/26)	7.7% (2/26)		
Positive	14	—	50% (7/14)	50% (7/14)	0.008	0.002
						
*ET*_*B*_*R*						
Negative	25	—	84% (21/25)	16% (4/25)		
Positive	14	7.1% (1/14)	50% (7/14)	42.9% (6/14)	0.056	0.065

a CR=complete response; PR=partial response; NC=no change. *P*^1^=CR *vs* PR *vs* NC (*χ*^2^ test). *P*^2^=response (CR+PR) *vs* nonresponse (NC) (*χ*^2^ test).

. Pathological response to chemotherapy was significantly decreased in ET_A_R-positive patients (*P*=0.002). Pathological NC was obtained in 50% of ET_A_R-positive patients as compared to 7.7% of ET_A_R-negative patients. None of the ET_A_R-positive patients had a pCR. Similar to ET_A_R, ET_B_R expression correlated with nonresponse (42.9%), although statistical significance was not reached (*P*=0.065). No significant correlation was observed for ET-1 expression and pathological response

#### Predictive value of ET axis expression for response

Multivariate analysis was performed by logistic regression to evaluate the predictive role of ET-1, ET_A_R, and ET_B_R status for clinical and pathological response. Clinicopathological factors associated with prognosis such as tumour size, lymph node status, grading, and steroid hormone receptor status were included for analysis. None of the investigated factors, ET-1, ET_A_R, or ET_B_R, had a predictive value for *clinical* response. Conversely, ET_A_R expression retained independence in predicting response, showing a significant inverse association with *pathological* response to treatment (*P*=0.009). Neither ET-1 (*P*=0.403) nor ET_B_R (*P*=0.056) expression was a significant obstacle to pathological response.

#### Treatment arms

Statistical analysis stratified for both treatment arms showed no differences between arm A and arm B concerning distribution of histopathological characteristics, expression of ET axis and response rates to chemotherapy.

## DISCUSSION

The indication for treating breast cancer with chemotherapy is generally based on clinical and tumorbiological characteristics such as lymph node involvement, hormone receptor status, and Her-2/neu expression, which are indicators of prognosis but do not necessarily indicate likelihood of response. To date, there is no reliable marker for the prediction of chemotherapy response in breast cancer patients to facilitate individualised treatment. This study highlights the potential clinical role of ET_B_R and especially of ET_A_R as predictors of response to neoadjuvant chemotherapy in patients with locally advanced breast cancer.

Previously, we have demonstrated that elevated ET-1, ET_A_R, and ET_B_R expression is more common in breast cancers of patients with diminished disease-free and overall survival. In that study we also observed a close correlation between ET_A_R-positive tumours and clinicopathological markers for poor prognosis (Wülfing *et al*, 2003). To assess whether the ET axis may influence breast cancer response to chemotherapy treatment, a series of patients with locally advanced breast cancer was evaluated.

In this study, we found that overexpression of the ET receptors was correlated with pathological NC following chemotherapy, thus providing evidence that overexpression of the ET receptors may adversely affect response to chemotherapy treatment. Higher pathological response rates in ET_B_R- and especially in ET_A_R-negative than in ET receptor-positive patients further support the hypothesis that ET receptor expression is associated with resistance to chemotherapy. Our data also suggest a potential ability of ET receptor expression to predict breast cancer susceptibility to chemotherapy. The independent predictive value of ET_A_R expression for pathological response was confirmed applying logistic regression. Similar to these findings, analysis of clinical response to neoadjuvant chemotherapy with respect to ET expression revealed a reduced effect in ET_A_R-positive patients as compared to ET_A_R-negative patients although differences between these groups were less obvious. To determine whether our results were merely a reflection of differences between distinct groups, we evaluated whether tumorbiological factors with well-established prognostic relevance were related to ET-1, ET_A_R, and ET_B_R expression. Except for a statistically significant inverse relationship between ET-1 expression and steroid hormone receptor status as well as between ET_A_R expression and proliferation index (Ki67), with respect to conventional prognostic markers no further significant correlations were found.

Accumulating data have shown that ET-1 acting through ET_A_R functions as a survival factor for carcinoma cells. In colon carcinoma cells, ET-1 inhibits apoptosis mediated by Fas-ligand (FasL), which induces cell death via caspase activation, or Paclitaxel (Eberl *et al*, 2000a, 2000b). Similarly, in ovarian carcinoma cells, paclitaxel-induced apoptosis is inhibited by ET-1 (via ET_A_R), triggering antiapoptotic signalling through *bcl-2*-dependent and phosphatidylinositol 3-kinase-mediated *AKT* pathways (Vacca *et al*, 2000). Conversely, cervical cancer cell growth *in vivo* could be stopped by ET_A_R blockade with Atrasentan (ABT-627), a selective ET_A_R-inhibitor, alone, and in that study Atrasentan displayed additive antitumour effects when administered in combination with the cytotoxic agent Paclitaxel (Bagnato *et al*, 2002). Also, in ovarian carcinoma xenograft models, inhibition of tumour growth by Atrasentan was found to be as effective as Paclitaxel (Rosano *et al*, 2003). Since it is conceivable that antiapoptotic factors may contribute to resistance to antitumoral therapy, the observed chemotherapy resistance in ET receptor overexpressing tumours may be attributed to the autocrine influence of ET-1 acting via ET_A_R.

From the evidence to date, it appears that ET-1 and ET_A_R play the predominant role in malignancies. Also, selective ET_A_R antagonism provides the most likely method of ET axis inhibition in cancer (Grant *et al*, 2003; Nelson *et al*, 2003). Our findings may reflect the pivotal role of ET_A_R alteration in breast cancer. The observed higher influence of ET_A_R rather than ET_B_R expression on chemotherapy response can be explained in terms of data that ET-1 signalling through ET_A_R imparts a survival benefit of carcinoma cells by inhibiting chemotherapy induced apoptosis. In contrast, so far, there is no evidence from the literature that ET_B_R signalling is also associated with apoptosis inhibition.

We suggest that in breast cancer ET-1 produced by the tumour cells acts in an autocrine mechanism via ET receptors. The lack of correlation between ET-1 expression and chemotherapy response could be explained by the fact that ET-1 exerts its effects through ET receptor binding. ET-1 levels and expression may, however, not be an appropriate correlate for the ET-1 activity at the microenvironment level. ET-1 expression may therefore not be an adequate indicator of the activity of the ET_A_R signalling. It is the expression of the ET_A_R that signifies the potential antiapoptotic effects of ET-1. Also in view of the literature, ET_A_R activation rather than ET-1 itself promotes tumour progression by means of various mechanisms (Nelson *et al*, 2003).

Our data support the predictive role of ET_A_R overexpression as a marker of adverse pathological response to chemotherapy treatment in breast cancer. However, generalisation of our results may be limited by the relatively small sample size. This limitation notwithstanding, our data could offer a valid background for further confirmatory research.

In conclusion, our findings suggest that increased expression of the ET receptors and especially of ET_A_R in breast carcinomas is associated with resistance to chemotherapy. Furthermore, in this cohort of patients with advanced breast cancer, the ET_A_R status was an independent predictor of response to chemotherapy. Thus, immunohistochemical analysis of ET_A_R expression may serve as a convenient, low-cost technique for identifying breast cancer patients with a poor chance of response to chemotherapy and, therefore, potential candidates for more individualised treatments. Since our findings can possibly be explained by an ET_A_R-mediated chemotherapy resistance, combining ET_A_R antagonism with conventional chemotherapy may improve the outcome of breast cancer patients.
